# Increased expression and retention of the secretory chaperone proSAAS following cell stress

**DOI:** 10.1007/s12192-020-01128-7

**Published:** 2020-07-20

**Authors:** Manita Shakya, Taha Yildirim, Iris Lindberg

**Affiliations:** Department of Anatomy and Neurobiology, University of Maryland School of Medicine, 20 Penn St, HSF2, S267, Baltimore, MD 21201 USA

**Keywords:** Cell stress, Secretory chaperones, proSAAS, Cell viability, *PCSK1N*

## Abstract

The secretory pathway of neurons and endocrine cells contains a variety of mechanisms designed to combat cellular stress. These include not only the unfolded protein response pathways but also diverse chaperone proteins that collectively work to ensure proteostatic control of secreted and membrane-bound molecules. One of the least studied of these chaperones is the neural- and endocrine-specific molecule known as proSAAS. This small chaperone protein acts as a potent anti-aggregant both *in vitro* and *in cellulo* and also represents a cerebrospinal fluid biomarker in Alzheimer’s disease. In the present study, we have examined the idea that proSAAS, like other secretory chaperones, might represent a stress-responsive protein. We find that exposure of neural and endocrine cells to the cell stressors tunicamycin and thapsigargin increases cellular proSAAS mRNA and protein in Neuro2A cells. Paradoxically, proSAAS secretion is inhibited by these same drugs. Exposure of Neuro2A cells to low concentrations of the hypoxic stress inducer cobalt chloride, or to sodium arsenite, an oxidative stressor, also increases cellular proSAAS content and reduces its secretion. We conclude that the cellular levels of the small secretory chaperone proSAAS are positively modulated by cell stress.

## Introduction

Neurons and endocrine cells represent specialized secretory cells characterized by the presence of a regulated secretory pathway, designed to accommodate the changing physiological need for secretion of either neuropeptide neurotransmitters or peptide hormones, respectively. Specialized proteins common to both tissue types include trafficking proteins involved in secretory granule formation and release and proteins involved in secretory protein processing and homeostasis.

Among this group of stored secretory proteins are two interesting small protein chaperones, namely, 7B2 (Seidah et al. 1983) and proSAAS (Fricker et al. 2000). Initially identified as neuroendocrine-specific proprotein convertase binding proteins (Braks and Martens 1994; Fricker et al. 2000), both 7B2 and proSAAS have been classed as members of the granin family of proteins (Huttner et al. 1991), though they lack the characteristic heavy glycosylation of all other granin family members and are significantly smaller. Both 7B2 and proSAAS contain proline-rich regions, at least one furin cleavage site, and are expressed almost exclusively in neural and endocrine tissues (Iguchi et al. 1984; Lanoue and Day 2001; Morgan et al. 2005). The carboxy-terminal peptide fragments of 7B2 and proSAAS are potent inhibitors of the proprotein convertases PC2 and PC1/3, respectively (Cameron et al. 2000; Fricker et al. 2000; Martens et al. 1994), enzymes which mediate the proteolytic cleavage of peptide precursors in the secretory pathway (reviewed in (Steiner 1998)). The about 20 kDa amino-terminal domains of both 7B2 and proSAAS have been shown to carry out chaperone anti-aggregant functions, both for convertases (Fortenberry et al. 2002; Lee and Lindberg 2008) and for other aggregating proteins (Helwig et al. 2013; Hoshino et al. 2014; Jarvela et al. 2016).

ProSAAS, encoded by the mouse gene *Pcsk1n*, is a 225-residue polypeptide originally discovered via mass spectroscopic screening of peptides in the brains of Cpe^fat^/Cpe^fat^ mice (Che et al. 2001; Fricker et al. 2000). The broad neuronal distribution of proSAAS, wider than that of PC1/3 (Feng et al. 2002; Lanoue and Day 2001; Morgan et al. 2005), supports the idea that proSAAS has physiological functions other than its interaction with PC1/3. The level of proSAAS is increased in the brains of rodents subjected to hypoxic stress and/or dehydration, implying a possible role in cell stress homeostasis (Gouraud et al. 2007; Mihailova et al. 2008; Wang et al. 2007).

Neurodegenerative diseases are known to involve the increased production of aggregating proteins, suggesting a lack of proteostatic control during disease progression. In accordance with the idea that proSAAS plays a role in brain proteostasis, several groups have identified immunoreactive proSAAS within neurofibrillary tangles and neuritic plaques in brain tissue obtained from patients with Alzheimer’s disease (AD), Pick’s disease, and Parkinsonism-dementia complex (Helwig et al. 2013; Kikuchi et al. 2003; Wada et al. 2004). Eight independent proteomic studies have identified proSAAS as a cerebrospinal fluid candidate biomarker in AD and/or frontotemporal dementia (Abdi et al. 2006; Choi et al. 2013; Davidsson et al. 2002; Finehout et al. 2007; Holtta et al. 2015; Jahn et al. 2011; Spellman et al. 2015; Wang et al. 2016), and recent transcriptomics studies have shown that brain proSAAS expression increases during Alzheimer’s progression (Mathys et al. 2019). Supporting its role as a neuronal chaperone in neurodegeneration, studies *in vitro* and in cell lines have demonstrated that proSAAS exhibits potent chaperone activity; can inhibit the fibrillation of beta amyloid, islet amyloid polypeptide, and α-synuclein at low stoichiometric ratios; and can protect cells from oligomer-induced cytotoxicity (Hoshino et al. 2014; Jarvela et al. 2016; Peinado et al. 2013). Collectively, these studies provide strong evidence to support the idea that brain proSAAS is involved in neuronal proteostasis.

In the current study, we have investigated the hypothesis that stressful conditions within the cell, and particularly within the endoplasmic reticulum (ER), might result in increased cellular levels of proSAAS. In the work described here, we have used primary neurons as well as endocrine and neuronal lines to investigate the relationship between cell stress and proSAAS expression.

## Methods

### Materials

Tunicamycin and thapsigargin were obtained from Sigma-Aldrich (St. Louis, MO), as were cobalt chloride (CoCl_2_) and sodium meta-arsenite (NaAsO_2_). The WST-1 cell proliferation reagent was purchased from Sigma-Aldrich. Cell culture media and supplements were obtained from Invitrogen (Carlsbad, CA). All oligonucleotides were synthesized by IDT (Rockville, MD).

### Cell culture and treatment

Neuro2A cells were purchased from the American Type Culture Collection (ATCC, Manassas, VA), while AtT-20 cells were obtained from the Mains laboratory (University of Connecticut, CT). AtT-20 cells were maintained in high glucose DMEM medium supplemented with 10% fetal bovine serum (FBS) and 10% Corning Nuserum (VWR, Bridgeport, NJ), while Neuro2A cells were grown in high glucose DMEM:Opti-MEM (1:1) medium supplemented with 5% FBS. All cells were maintained at 37 °C in a humidified atmosphere containing 5% CO_2_. Growth media were then replaced with prewarmed OptiMEM plus 0.1% aprotinin (Sigma-Aldrich, St. Louis, MO) for 1 h prior to the start of the treatment. All the treatments were prepared in prewarmed OptiMEM plus 0.1% aprotinin.

### Generation of proSAAS-overexpressing AtT-20 cell clones

Approximately 1 × 10^6^ cells were plated into 10-cm dishes and transfected the following day with cDNA encoding mouse proSAAS (Fortenberry et al. 2002) using Lipofectin (Invitrogen). Cells were selected with 100 μg/ml hygromycin (Sigma-Aldrich). After 4 weeks, hygromycin-resistant clones were picked using the agarose overlay method (https://www.youtube.com/watch?v=IhOP397sCC8) and subcloned into 24-well plates. Screening of overnight-conditioned OptiMEM was accomplished using proSAAS radioimmunoassay (Sayah et al. 2001), and the three highest-expressing clones were saved for use in these experiments. Western blotting confirmed proSAAS overexpression relative to actin expression.

### Generation of CRISPR/Cas9-generated Pcsk1n-knockout AtT-20 cell clones

AtT-20 cells were transfected with the p459x plasmid (Addgene, Rockville, MD; plasmid #62988) encoding one of two double-stranded synthetic mouse proSAAS guide RNAs (either GCACCAAAATGCCGACGCCCC or CTGCCCCCCACCCTGTCAGCG). All construct sequences were verified by sequence analysis, performed at the University of Maryland Genomics Core Laboratories. Cells were then treated with 2 μg/ml puromycin; 3 weeks later, RNA was prepared from individual clones, and knockouts were selected using RT-PCR using primers flanking the proSAAS sequence. Western blotting was used to confirm the loss of proSAAS expression in three independent clones.

### Primary neuron cultures

Primary neurons from either the hippocampus or the cerebral cortex of E17 rat embryos were cultured as described previously (Frost et al. 2010). Briefly, hippocampi or small pieces of frontal cerebral cortex were cut into small pieces, digested with 0.25% trypsin-EDTA, and mechanically dissociated by gentle pipetting with a series of small-bore Pasteur pipettes. Cells were resuspended in Neurobasal medium (Invitrogen) supplemented with 5% B27 supplement, 5% penicillin-streptomycin, and 2 mM L-GlutaMAX (all from Invitrogen) and then cultured in 12- or 24-well plates pre-coated with poly-L-lysine at 37 °C and 5% CO_2_ in a humidified atmosphere. Primary neurons were cultured for 10–14 days prior to treatment with various concentrations of tunicamycin or thapsigargin (Sigma-Aldrich), as described in each experiment.

### Induction of cellular stress by heat shock

Heat shock stress was induced by floating a tissue culture plate containing cells, covered and sealed with parafilm, in a 42 °C water bath for 1 h followed by a recovery period of 12 h at 37 °C in a 5% CO_2_ humidified incubator.

### RNA isolation, reverse transcription, and real-time polymerase chain reaction

Total RNA was isolated from each well of a 12-well plate using a Direct-Zol RNA Mini Kit (Zymo Research, Irvine, CA). The concentration and purity (A260/A280 ratio) of RNA were determined using a NanoDrop 2000 spectrophotometer. First-strand complementary deoxyribonucleic acid (DNA) was synthesized from 0.5 μg of total RNA using an iScript cDNA synthesis kit (Bio-Rad Laboratories; Richmond, VA). The real-time polymerase chain reaction (RT-PCR) was performed in 10 μl reaction volume containing 5 ng cDNA, power SYBR Green PCR master mix (Applied Biosystems, Warrington, UK), and 500 nM each of specific forward and reverse primer using CFX96 RT-qPCR system (Bio-Rad) with the following cycle conditions (initial denaturation at 95 °C for 3 min, and 39 cycles of denaturation at 95 °C for 15 s with annealing at 60 °C for 15 s and extension at 72 °C for 30 s). Melting peaks were determined with melting curve analysis to ensure amplification of a single product. All reactions were performed in triplicate and relative mRNA levels compared and expressed as fold of control level (2^−DDCt^ method) using glyceraldehyde 3-phosphate dehydrogenase (GAPDH) as internal controls. The following primers were purchased from IDT and used for RT-PCR: GAPDH forward, 5′-CGTGTTCCTACCCCCAATGT-3′, GAPDH reverse, 5′-TGTCATCATACTTGGCAGGTTTCT-3′; proSAAS forward, 5′-CTGTGGACCAGGATTTGGGT-3′, proSAAS reverse, 5′-TTTGACGCGTAGCAGAGC-3′; and BiP forward, 5′-AGGATGCGGACATTGAAGACTTTA-3′ and BiP reverse, 5′-TCCACTTCCATAGAGTTTGCTGATA.

### Conditioned media protein precipitation

Conditioned media samples were mixed with twice their volumes of cold methanol and 2 μg of glycogen (molecular biology grade, catalog no. R0561, Thermo Scientific), vortexed, and incubated overnight at 4 °C. Proteins were precipitated by centrifugation at 12000 rpm for 10 min at 4 °C. The supernatant was carefully decanted without dislodging the protein pellet. The pellets were air-dried in a chemical hood for 20–30 min, then mixed with 2X Laemmli sample buffer containing 6 M urea, vortexed, and boiled at 95 °C for 5 min prior to gel loading.

### Western blotting

Cells were washed with Opti-MEM and then directly extracted by application of 2X Laemmli sample buffer. Following boiling at 95 °C for 5 min, samples were sonicated to reduce viscosity. Proteins were separated by SDS-PAGE using precast Novex™ NuPAGE 14% gels (Fisher Scientific) or 15% Tris-HCl Criterion gels (Bio-Rad) for proSAAS and AnyKD gels (Bio-Rad) for BiP. Fifteen percent of cell lysate samples and 50% of the concentrated conditioned medium samples were loaded onto pre-cast 14 or 15% Tris-HCl gels (Bio-Rad) to blot for proSAAS; to blot for BiP, 5% of cell lysate was loaded onto AnyKD gels (Bio-Rad). Proteins were then electroblotted onto nitrocellulose membranes using a semi-dry Bio-Rad Transblot apparatus (25 V for 7 min for proSAAS and 25 V for 10 min for BiP). Before immunoblotting with proSAAS, the membrane was cross-linked with 1% glutaraldehyde in phosphate-buffered saline (PBS) for 15 min, after which it was washed thoroughly (5 times for 5 min) with PBS to remove glutaraldehyde. This cross-linking step was found to radically increase proSAAS antiserum reactivity. Membranes were then blocked for 1 h at room temperature with blocking buffer (5% blocking-grade milk (Bio-Rad) in Tris-buffered saline (TBS) containing 0.05% (v/v) Triton X-20). Primary antisera used for each blot were resuspended in the same buffer and consisted of rabbit proSAAS antisera LS45 and LS46, raised against recombinant His-tagged proSAAS 1–180 (Iris Lindberg, University of Maryland, Baltimore (Hoshino et al. 2014; Jarvela et al. 2016)). These two antisera recognize intact proSAAS as well as various carboxy- and amino-terminally truncated forms. A Protein A-agarose column (Invitrogen) was used to purify the IgG fraction from LS46 prior to immunoblotting; between 5 and 6 μg/ml was used for blotting.

Other antisera used in this study include a mouse monoclonal antibody to actin (Sigma-Aldrich; catalog no. A2228; RRID, AB_476697) and a rabbit polyclonal antibody to BiP/GRP78 (Abcam; ab21685; RRID, AB_2119834), each at dilutions of 1:5000. Horseradish peroxidase–tagged proteins were visualized using the Clarity substrate (Bio-Rad Laboratories) following incubation with secondary horseradish peroxidase–linked antibody directed against either rabbit IgG (Jackson ImmunoResearch Laboratories, West Grove, PA; 1:5000; RRID, AB_2307391) or mouse IgG (Bio-Rad Laboratories; 1:5000; RRID, AB_11125547). Quantitation of bands was performed on a Bio-Rad Imager using the ImageLab 6.0 program.

### Cell viability assay

About 50,000 cells were plated per well in a 48-well plate in a final well volume of 300 μl. Cells were treated with fresh medium with or without 0.25/0.5 μg/ml of tunicamycin (Sigma) for 24 h. The medium was then replaced with fresh medium containing a 1:10 dilution of WST-1 (Sigma) and the plate incubated at 37 °C for 20–30 min. After incubation, 100 μl of each well was transferred to a 96-well plate and the absorbance at 450 nm was measured; WST-containing blank medium was subtracted from all values. The value for vehicle-treated cells was set as 100%.

### Oxidative stress assay

Intracellular reactive oxygen species (ROS) were measured using the cell permeable reagent 2′,7′-dichlorofluorescein diacetate (DCFDA) (ab113851, Abcam, Cambridge, MA, USA) according to the manufacturer’s instructions. After diffusion into the cell, DCFDA is deacetylated by cellular esterases to a non-fluorescent compound, which is later oxidized by ROS into 2′,7′-dichlorofluorescein (DCF). Briefly, Neuro2A cells were plated at a density of 25,000 cells/well in 96-well optical black plates. The culture medium was removed, and the cells were stained with 20 μM DCFDA diluted in kit buffer for 45 min in the dark at 37 °C. Cells were then treated with either water or 0.5 mM sodium meta-arsenite (NaAsO_2_) and incubated for 30 min. End-point fluorescence from 6 replicate wells for each experimental condition was measured in a fluorescence microplate reader (SpectraMax M2, Molecular Devices) at 485 nm excitation and 535 nm emission; background fluorescence from blank medium without DCFDA was subtracted from all values.

### Statistical analysis

Statistical analyses were performed using GraphPad Prism 8. Results are expressed as mean ± SD. Multiple comparisons between the different groups were conducted using one-way ANOVAs, two-way ANOVAs, and Student’s *t* test, as described in the legends. Differences were considered statistically significant when *p* < 0.05.

## Results

### Tunicamycin-induced ER stress increases proSAAS expression

**Fig. 1:**
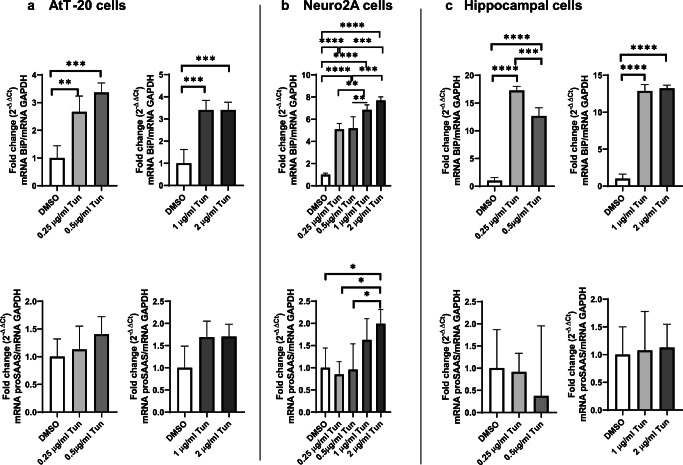
ER stress induced by tunicamycin increases the expression of BiP and proSAAS mRNAs. Tunicamycin (Tun) exposure increases mRNA levels of the ER stress marker BiP in AtT-20 cells (**a**), Neuro2A cells (**b**), and primary hippocampal neurons (**c***)* but increases proSAAS mRNA levels only in Neuro2A cells*.* Cells were treated with increasing concentrations of tunicamycin (Tun) for 24 h, and mRNA levels were determined by quantitative real-time PCR. Each mRNA is shown relative to GAPDH and plotted as the fold increase compared with the vehicle control (DMSO). The data shown represent the averages of four biological and three technical replicates ± SD. *p ≤ 0.05; **p ≤ 0.01; ***p ≤ 0.001; ****p ≤ 0.0001; one-way ANOVA with the Tukey post hoc test

To determine proSAAS responsivity to ER stress, we investigated the protein and mRNA levels of proSAAS in endocrine and neuronal cell lines treated with two different ER stress inducers: tunicamycin and thapsigargin. We first treated cells with increasing concentrations of the N-glycosylation inhibitor tunicamycin for 24 h. Induction of ER stress by tunicamycin is known to upregulate BiP/GRP78, an ER-resident chaperone and the most commonly used marker for unfolded protein response (UPR) activation (Hendershot 2004). In agreement, we found robust upregulation of BiP mRNA in AtT-20 (Fig. 1c; top row), Neuro2A (Fig. 1b; top row), and hippocampal cells (Fig. 1c; top row) with all tested doses of tunicamycin. In these same samples, we also found modest tunicamycin-induced upregulation of proSAAS mRNA in Neuro2A cells at the higher tunicamycin concentrations (1 and 2 μg/ml) (Fig. 1b; bottom row); sample variability (likely due to low proSAAS expression) precluded our ability to make statistical conclusions as regards AtT-20 cells (Fig. 1a; bottom row) and and hippocampal cells (Fig. 1c; bottom row).

**Fig. 2:**
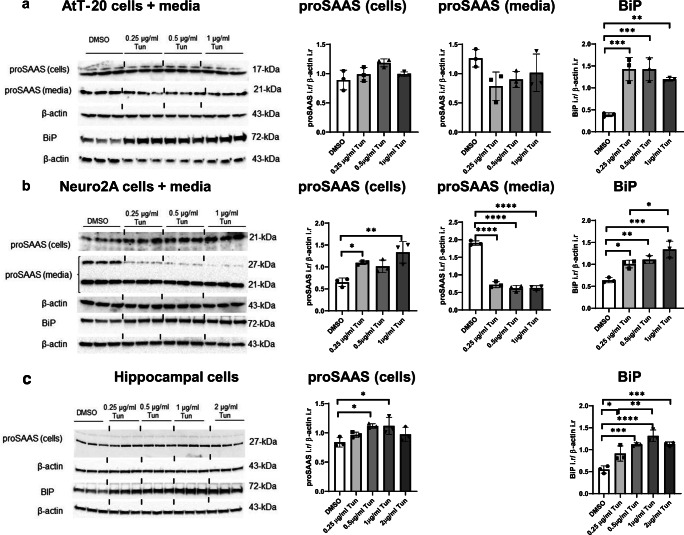
ER stress induced by tunicamycin increases cellular BiP and proSAAS protein while suppressing proSAAS secretion. Immunoblot of cell lysate and conditioned media collected from AtT-20 cells (**a**), Neuro2A cells (**b**), and primary hippocampal neurons (**c**) treated with increasing concentrations of tunicamycin (Tun) for 24 h. Immunoblots probed with BiP and proSAAS antiserum were normalized to β-actin as a loading control. Data are presented as mean ± SD of three replicates. *p ≤ 0.05; **p ≤ 0.01; ***p ≤ 0.001; ****p ≤ 0.0001; one-way ANOVA with the Tukey post hoc test. i.r., immunoreactivity

We then performed Western blot analyses to determine whether ER stress alters proSAAS expression at the protein level. Consistent with mRNA results, all tested doses of tunicamycin caused robust upregulation of BiP protein expression. We observed a trend of a modest increase in cellular proSAAS protein levels in all cell types, but this reached statistical significance only in Neuro2A (Fig. 2b) and hippocampal cells (Fig. 2c). Since proSAAS is a secretory chaperone, we next examined how ER stress affects its secretion. A Western blot of conditioned media showed two major bands of proSAAS under reducing conditions: the upper band at 27 kDa likely represents intact proSAAS, and the lower at 17 kDa band represents a cleaved form (Fricker et al. 2000; Sayah et al. 2001). The 27 kDa unprocessed form was not detectable in media or lysate obtained from AtT-20 cells, most likely due to the abundant expression of the prohormone convertase PC1/3 in this cell line (Fricker et al. 2000; Hornby et al. 1993; Sayah et al. 2001); primary hippocampal cells were consistently unable to process 27 kDa proSAAS. Interestingly, proSAAS secretion was profoundly reduced in all cell types following tunicamycin treatment; in Neuro2A cells, the intact, 27 kDa form of proSAAS, was the most strongly reduced form. We were not able to detect proSAAS in the conditioned medium of hippocampal neurons, most likely due to the low rate of basal secretion from these well-differentiated cells **(**Fig. 2c**)**.

### Stable upregulation of proSAAS exerts a protective effect against tunicamycin-induced cell stress in AtT-20 cells, while proSAAS knockout sensitizes cells to tunicamycin

**Fig. 3:**
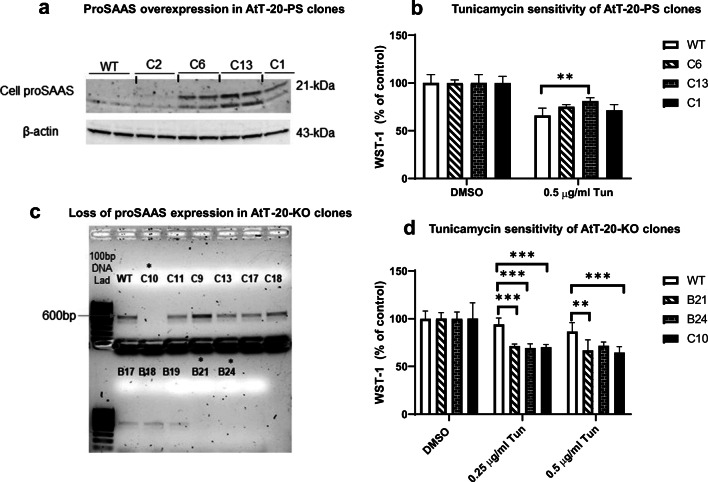
ProSAAS overexpression decreases tunicamycin sensitivity, while proSAAS knockout increases sensitivity. **a** Overexpression of proSAAS in three AtT-20 cell clones was verified by immunoblotting; results were normalized to actin. **b** Wild-type and proSAAS-overexpressing clones were treated with 0.5 μg/ml of tunicamycin (Tun) or vehicle for 24 h and cell viability assessed. **c** Loss of proSAAS expression in AtT-20-KO cell clones is shown by PCR following reverse transcription of mRNA as the loss of about 600 bp proSAAS PCR product. **d** Wild-type and knockout clones were treated with 0.25 μg/ml and 0.5 μg/ml of tunicamycin (Tun) or vehicle for 24 h and cell viability assessed. WST-1 results are expressed as percentages of corresponding non-Tun-treated control cells*.* Data are presented as mean ± SD of six replicates. *p ≤ 0.05; **p ≤ 0.01; ***p ≤ 0.001; ****p ≤ 0.0001; two-way ANOVA with the Tukey post hoc test

Since our findings indicated that ER stress results in increased cellular proSAAS protein and decreased secretion, we investigated whether intracellular accumulation of proSAAS might have functional implications in the cellular protection against ER stress. To explore this idea, wild-type AtT-20 cells or clones stably overexpressing proSAAS (Fig. 3a) were treated with either vehicle or 0.5 μg/ml of tunicamycin for 24 h, and cell viability was quantified using WST-1, an assay which measures mitochondrial activity (Fig. 3b). We observed that proSAAS overexpression produced limited rescue of cells from tunicamycin toxicity, with mitochondrial activity increasing by about 5%, 10%, and 15%, respectively, in the three proSAAS-overexpressing clones as compared with the wild-type control. Interestingly, the extent of protection (Fig. 3b) appeared to be correlated with the level of clonal proSAAS expression (Fig. 3a). While these tunicamycin-induced differences were small, similar results were obtained in two additional independent experiments.

In order to further substantiate the protective role of proSAAS under ER stress conditions, we tested the sensitivity to ER stress of stable AtT-20 clones in which proSAAS expression was knocked out using CRISPR/Cas9-mediated gene deletion. ProSAAS knockout clone lines B and C were generated using double-stranded synthetic mouse proSAAS guide RNAs (either GCACCAAAATGCCGACGCCCC or CTGCCCCCCACCCTGTCAGCG). Loss of proSAAS expression was determined by PCR of reverse-transcribed RNA (Fig. 3c), and three independent clones, B21, B24, and C10, showing complete loss of proSAAS, were selected. A WST-1 assay demonstrated that proSAAS knockout sensitized these three clones to tunicamycin, potentiating cell death by about 20% (Fig. 3d). Taken together, these results support the idea that proSAAS expression is correlated with a modest cytoprotective effect against tunicamycin.

### Cell stress induced by thapsigargin increases the levels of proSAAS

**Fig. 4:**
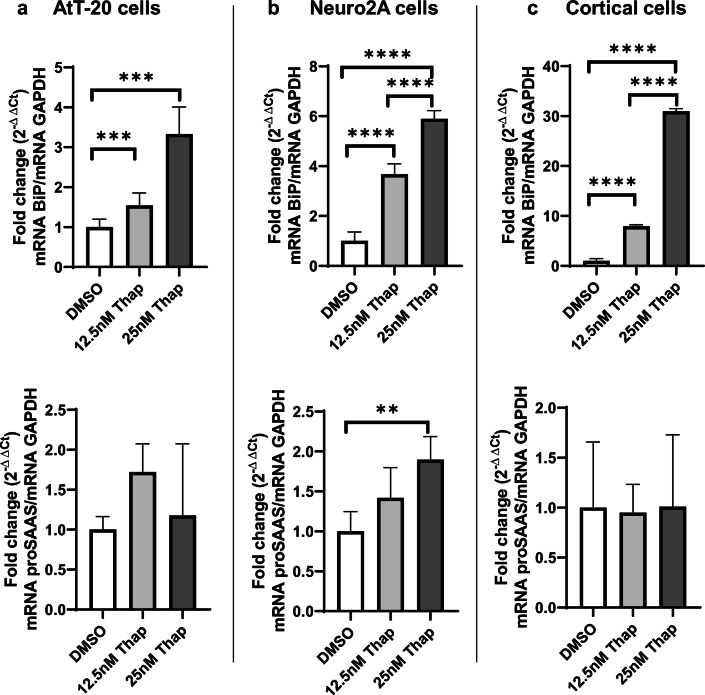
ER stress induced by thapsigargin increases the expression of BiP and proSAAS mRNAs. The effect of the ER calcium stressor thapsigargin (Thap) on mRNA levels of BIP and proSAAS in AtT-20 cells (**a**) and Neuro2A (**b**) cells and primary cortical cells (**c**). Cells were treated with increasing concentrations of thapsigargin for 24 h, and mRNA levels were determined by quantitative real-time PCR. Each mRNA is shown relative to GAPDH and plotted as the fold increase compared with the vehicle control (DMSO). The data shown represent the averages of four biological and three technical replicates ± SD. *p ≤ 0.05; **p ≤ 0.01; ***p ≤ 0.001; ****p ≤ 0.0001; one-way ANOVA with the Tukey post hoc test

The above data show that tunicamycin-induced cell stress increases cellular proSAAS protein levels and decreases its secretion. Various ER stressors perturb ER homeostasis in different ways; thus, we tested another well-known but mechanistically distinct ER stressor, thapsigargin, which induces the UPR by blocking the sarcoplasmic/endoplasmic reticulum Ca^2+^ ATPase (Oslowski and Urano 2011). Cells were treated with a low dose of thapsigargin for 24 h in order to induce ER stress without significantly affecting cell viability. As seen with tunicamycin, robust induction of BiP mRNA was induced by this compound in all three cell types (Fig. 4, top panels). Levels of proSAAS mRNA exhibited a lesser, but still significant, increase in Neuro2A cells, though not in other cell types, possibly due to the relatively large variability (Fig. 4, bottom panels).

**Fig. 5:**
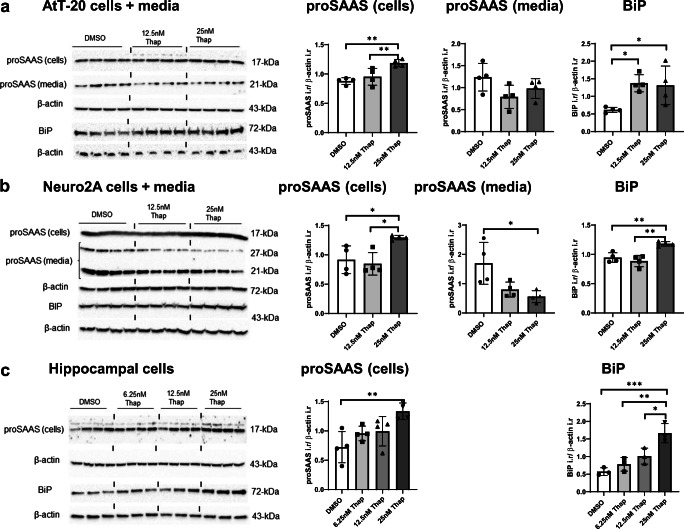
ER stress induced by thapsigargin increases cellular BiP and proSAAS levels while suppressing proSAAS secretion. AtT-20 cells (**a**), Neuro2A cells (**b**), and primary cortical cells (**c**) were exposed to thapsigargin (Thap) for 24 h, and cellular lysates and the conditioned medium were assessed for proSAAS expression (hippocampal medium proSAAS was below the limit of detection and is thus not shown). Immunoblots probed with BiP and proSAAS antiserum were normalized to β-actin as a loading control. Data are presented as mean ± SD of four replicates. *p ≤ 0.05; **p ≤ 0.01; ***p ≤ 0.001; ****p ≤ 0.0001; one-way ANOVA and with the Tukey post hoc test. i.r., immunoreactivity

Cellular proSAAS protein levels were increased following thapsigargin treatment in all three cell types tested, in parallel with BiP protein levels (Fig. 5a–c). Similar to tunicamycin, thapsigargin treatment significantly reduced secreted proSAAS levels in conditioned media obtained from Neuro2A and AtT-20 cells (Fig. 5a–c); again, it was not possible to measure proSAAS in hippocampal cell-conditioned medium as it was below the limit of detection. Collectively, these data support the idea of robust retention of proSAAS following exposure to ER stressors.

### Sodium arsenite-induced oxidative cell stress and CoCl_2_-induced hypoxic stress both increase proSAAS expression in Neuro2A cells

**Fig. 6:**
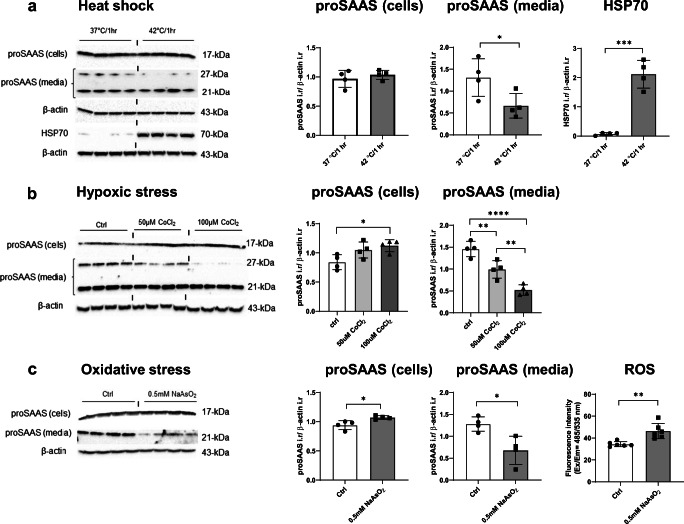
Oxidative stress induced by sodium arsenite and hypoxic stress induced by cobalt chloride increase the expression of proSAAS and reduce its secretion. Neuro2A cells were subjected to a short heat stress (**a**) or treated with increasing concentrations of cobalt chloride (**b**) for 24 h or 0.5 mM sodium arsenite for 30 min (**c**). Immunoblots of cell lysates and secretion medium were probed with proSAAS antiserum and normalized to β-actin as a loading control. Blotting with HSP70 antiserum, normalized to β-actin, was used as a positive marker for heat shock stress (**a**). The presence of oxidative stress was confirmed by direct measurement of ROS levels (**c**). Data are presented as mean ± SD of four replicates + SD. *p ≤ 0.05; **p ≤ 0.01; ***p ≤ 0.001; ****p ≤ 0.0001; one-way ANOVA with the Tukey post hoc test and Student *t* test. i.r., immunoreactivity

We next asked which other kinds of cellular stressors might result in proSAAS upregulation. Cells were exposed to classic heat shock and oxidative and hypoxic stressors. Heat stress, accomplished by placing cells at 42 °C for 1 h, did not significantly elevate cellular proSAAS, but did increase expression of the control protein HSP70 as expected. Interestingly, the release of proSAAS was significantly reduced with heat shock treatment (Fig. 6a).

We induced hypoxic stress with CoCl_2_, which has been used as a chemical hypoxia-inducing agent for various types of neural cells (Gotoh et al. 2012). Exposure of Neuro2A cells to increasing concentrations of CoCl_2_ for 24 h resulted in a dose-dependent increase in cellular proSAAS levels, with a corresponding dose-dependent decrease in proSAAS secretion (Fig. 6b). We attempted to use the expression of hypoxia-inducible factor 1-alpha (HIF-1α) as a positive marker of hypoxic stress, since both 50 μM and 100 μM CoCl_2_ have been shown to induce robust induction of this protein (Zhi-Jun Dai et al. 2012); however, the levels of this transcription factor were too low to detect.

Lastly, oxidative stress was induced by a short exposure (30 min) of Neuro2A cells to 0.5 mM sodium arsenite (NaAsO_2_), a well-characterized oxidative stress-inducing agent and a promoter of stress granule assembly (Fazakerley et al. 2018). We first showed that arsenite exposure resulted in significant induction of intracellular ROS levels (Fig. 6c). Similar to ER stress and hypoxic stress, sodium arsenite treatment also resulted in significantly elevated cellular proSAAS and reduced proSAAS secretion (Fig. 6c). We thus conclude that cellular proSAAS retention is responsive not only to ER stressors but also to certain types of general cell stressors, but not to elevated temperature.

## Discussion

Cells are constantly exposed to physiological and environmental stresses that alter normal cell behavior and increase cell vulnerability. To cope with ongoing stress and return cells to homeostasis, various stress-responsive genes, proteins, and pathways are upregulated at the time of insult (Oakes 2020). Central to this cellular adaptation to stress is the expression of molecular chaperones, which protect intracellular proteins from misfolding or aggregation, inhibit cell death signaling cascades, and preserve intracellular signaling pathways (Oakes and Papa 2015; Voth and Jakob 2017). While the bulk of cellular chaperones are localized within the cytosol, professional secretory cells (such as endocrine cells and neurons) contain a large complement of lumenal chaperones in early secretory compartments which are required for secretory protein quality control (reviewed in (Liu et al. 2018)). Most of these secretory chaperones are retained with the ER and are neither trafficked through the secretory pathway nor packaged into secretory granules. Indeed, one of the only secreted chaperones known to be trafficked through the regulated secretory pathway is the small neuroendocrine-specific molecular chaperone proSAAS (Fortenberry et al. 2002; Wardman and Fricker 2014).

Though proSAAS was initially identified in 2000 (Fricker et al. 2000), its chaperone action was not recognized until 2014 (Hoshino et al. 2014), and our knowledge regarding its intracellular and extracellular functions is still incomplete. Prior studies have shown that proSAAS-encoding lentivirus blocks α-synuclein-induced cytotoxicity in primary cultures of nigral dopaminergic neurons (Jarvela et al. 2016), and recombinant proSAAS blocks α-synuclein-induced cytotoxicity in SH-SY5Y cells as well as the cytotoxic effect of Abeta 1-42 oligomers in Neuro2A cells (Hoshino et al. 2014). Over the past decade, cell injury secondary to chronic ER and oxidative stress has been increasingly implicated as a central contributor to the pathophysiology of a wide range of neurodegenerative diseases (Hetz and Mollereau 2014; Valenzuela et al. 2018). Therefore, we speculate that the neuroprotective function of proSAAS may arise as a portion of its general role in cytoprotection against ER and oxidative stressors.

In the present study, we set out to explore the idea that cellular proSAAS expression might be stress-responsive by examining the regulation of the proSAAS gene and protein expression following various stressors: ER stress, oxidative stress, hypoxia, and heat shock. We observed that cellular proSAAS protein levels increased in response to the ER stressors tunicamycin and thapsigargin. However, incubation of cells with these same ER stress inducers did not significantly affect the levels of proSAAS mRNA (except at high concentrations in Neuro2A cells). Thus, the increase in cellular proSAAS following ER stress likely does not depend on direct transcriptional activation.

Various types of ER stress clearly alter the cellular trafficking patterns of secretory proteins. Basal secretion of most secretory proteins is decreased in response to stress, most likely due to reduced translation resulting from activation of the unfolded protein response (Walter and Ron 2011); however, the secretion of certain ER proteins is increased in response to stress (Genereux et al. 2015; Henderson et al. 2013). We found that all of the cell stressors tested, i.e., ER stress, oxidative stress, hypoxic stress, and, surprisingly, even heat shock, sharply reduced the levels of proSAAS in the secretion medium; this was especially apparent in Neuro2A cells. This finding supports the idea that the increased accumulation of intracellular proSAAS results largely from decreased secretion. We attempted to gain information on the specificity of cellular retention of proSAAS by examining the secretion of endogenous secretogranin 2 in these same samples, but the results proved inconclusive. Increased cellular retention of secretory proteins following exposure to ER stressors (such as tunicamycin, which blocks N-linked sugar attachment) may occur as a result of misfolding; accumulated misfolded secretory proteins are then subjected to endoplasmic reticulum-associated degradation (ERAD) (Walter and Ron 2011). In this regard, it is important to note that proSAAS, a small protein which lacks both N-linked sugars and disulfide bonds, is predicted to have a largely disordered structure (Jarvela et al. 2016). It is interesting to note that ER stressors reduce extracellular secretion of the chaperone clusterin, which can then undergo stress-induced retrotranslocation into the cytosol (Li et al. 2013; Nizard et al. 2007). In preliminary confocal experiments, we could not detect a similar cytosolic retrotranslocation phenomenon for proSAAS; retained proSAAS appears to remain within the secretory pathway. Given the known anti-aggregant functions of proSAAS (Hoshino et al. 2014; Jarvela et al. 2016), we speculate that a stress-induced increase in cellular levels might represent an adaptive strategy to block protein aggregation within the secretory pathway.

The present results additionally demonstrate that cellular proSAAS protein levels also increased in response to hypoxic and oxidative stressors, while heat shock stress failed to increase cellular proSAAS. In agreement, the levels of proSAAS-derived fragments have been shown to be upregulated in hypothalamic extracts obtained from rats subjected to hypoxic stress (Mihailova et al. 2008). Clusterin expression has been also shown to be strongly stimulated by hypoxic stress (Kim et al. 2007), oxidative stress (Trougakos 2013; Viard et al. 1999), and heat shock (Michel et al. 1997; Viard et al. 1999). With regard to hypoxic stress, transcriptional activation rather than cellular retention appears to be the major mechanism for increased cellular clusterin, since clusterin secretion is also increased (Kim et al. 2007); however, during heat shock stress, decreases in cellular clusterin occur in parallel with increased secretion (Viard et al. 1999).

Why would cells choose to retain rather than to secrete secretory chaperones? In addition to a potential blockade of intralumenal secretory pathway aggregation, there is substantial evidence supporting prosurvival effects of chaperones in response to cell stress. Knockdown of clusterin increases the sensitivity of the HCC cell line to tunicamycin-induced apoptosis, whereas clusterin overexpression abolishes this effect (Wang et al. 2013). Likewise, silencing of the clusterin gene significantly enhances the sensitivity of cells to apoptotic death induced by heat shock and oxidative stress (Viard et al. 1999). To explore potentially similar cellular functions for proSAAS, we generated stable proSAAS knockout and proSAAS-overexpressing AtT-20 cell lines. ProSAAS knockout rendered cells more susceptible to tunicamycin stress-induced cytotoxicity, while overexpression of proSAAS improved cell viability. These data support the notion that accumulation of intracellular proSAAS may represent a physiological defense mounted by cells to reduce cell damage and maintain cell viability during periods of increased stress. In support of this idea, we have shown that proSAAS overexpression increases the viability of cells exposed to toxic levels of aggregating oligomers (Hoshino et al. 2014; Jarvela et al. 2016).

In conclusion, extending our earlier studies, we now report that proSAAS is a general stress-responsive protein. Future studies will focus on understanding the mechanisms underlying the cytoprotective functions of proSAAS by dissection of downstream signaling pathways.

## Data Availability

Original data and all new reagents will be made available upon request.

## Abbreviations

AD: Alzheimer’s disease
bp: Base pair
CSF: Cerebrospinal fluid
ER: Endoplasmic reticulum
FTD: Frontotemporal dementia
PC1/3: Proprotein convertase-1/3
DNA: Deoxyribonucleic acid
RT-PCR: Real-time polymerase chain reaction
GAPDH: Glyceraldehyde 3-phosphate dehydrogenase
PBS: Phosphate-buffered saline
TBS: Tris-buffered saline
ROS: Reactive oxygen species
DCFDA: 2′,7′-Dichlorofluorescein diacetate
HIF-1α: Hypoxia-inducible factor 1-alpha
UPR: Unfolded protein response
Tun: Tunicamycin
Thap: Thapsigargin
i.r.: Immunoreactivity
